# In a Concurrent Memory and Auditory Perception Task, the Pupil Dilation Response Is More Sensitive to Memory Load Than to Auditory Stimulus Characteristics

**DOI:** 10.1097/AUD.0000000000000612

**Published:** 2019-02-27

**Authors:** Adriana A. Zekveld, Sophia E. Kramer, Jerker Rönnberg, Mary Rudner

**Affiliations:** 1 Department of Behavioural Sciences and Learning, Linköping University, Linköping, Sweden; 2 Linnaeus Centre HEAD, The Swedish Institute for Disability Research, Linköping and Örebro Universities, Linköping, Sweden; 3 Section Ear & Hearing, Department of Otolaryngology-Head and Neck Surgery and Amsterdam Public Health research institute VU University Medical Center, Amsterdam, The Netherlands.

**Keywords:** Listening effort, Memory processing, Pupil dilation response, Speech perception

## Abstract

**Objectives:**

Speech understanding may be cognitively demanding, but it can be enhanced when semantically related text cues precede auditory sentences. The present study aimed to determine whether (a) providing text cues reduces pupil dilation, a measure of cognitive load, during listening to sentences, (b) repeating the sentences aloud affects recall accuracy and pupil dilation during recall of cue words, and (c) semantic relatedness between cues and sentences affects recall accuracy and pupil dilation during recall of cue words.

**Design:**

Sentence repetition following text cues and recall of the text cues were tested. Twenty-six participants (mean age, 22 years) with normal hearing listened to masked sentences. On each trial, a set of four-word cues was presented visually as text preceding the auditory presentation of a sentence whose meaning was either related or unrelated to the cues. On each trial, participants first read the cue words, then listened to a sentence. Following this they spoke aloud either the cue words or the sentence, according to instruction, and finally on all trials orally recalled the cues. Peak pupil dilation was measured throughout listening and recall on each trial. Additionally, participants completed a test measuring the ability to perceive degraded verbal text information and three working memory tests (a reading span test, a size-comparison span test, and a test of memory updating).

**Results:**

Cue words that were semantically related to the sentence facilitated sentence repetition but did not reduce pupil dilation. Recall was poorer and there were more intrusion errors when the cue words were related to the sentences. Recall was also poorer when sentences were repeated aloud. Both behavioral effects were associated with greater pupil dilation. Larger reading span capacity and smaller size-comparison span were associated with larger peak pupil dilation during listening. Furthermore, larger reading span and greater memory updating ability were both associated with better cue recall overall.

**Conclusions:**

Although sentence-related word cues facilitate sentence repetition, our results indicate that they do not reduce cognitive load during listening in noise with a concurrent memory load. As expected, higher working memory capacity was associated with better recall of the cues. Unexpectedly, however, semantic relatedness with the sentence reduced word cue recall accuracy and increased intrusion errors, suggesting an effect of semantic confusion. Further, speaking the sentence aloud also reduced word cue recall accuracy, probably due to articulatory suppression. Importantly, imposing a memory load during listening to sentences resulted in the absence of formerly established strong effects of speech intelligibility on the pupil dilation response. This nullified intelligibility effect demonstrates that the pupil dilation response to a cognitive (memory) task can completely overshadow the effect of perceptual factors on the pupil dilation response. This highlights the importance of taking cognitive task load into account during auditory testing.

## INTRODUCTION

Successful speech understanding in everyday listening conditions depends on a listener’s ability to cope with challenging auditory conditions (e.g., background noise or the effects of hearing loss) and to exploit any available contextual cues to compensate for difficulties in understanding speech (Miller & Isard 1963; Wingfield & Tun 2007; Zekveld et al. 2011b). In the current study, pupillometry was used to investigate the influence of acoustic degradation and semantic context (text cues) on cognitive processing load during a speech understanding task. Furthermore, we assessed the influence of sentence repetition and the semantic congruency of the cues and sentences on the recall of the contextual cues and processing load during recall. In the following sections, we explain our hypotheses after briefly introducing relevant concepts and background literature concerning (1) the use of contextual information to support speech understanding, (2) cognitive processing and effort during speech understanding, (3) recall, and (4) the relevance of cognitive abilities.

### Use of Contextual Information

We recently performed a series of studies to assess whether providing semantic context preceding the sentence (visual text cues consisting of three words that were semantically related to the content of the sentence) influenced how accurately listeners understood sentences presented in background noise at different signal to noise ratios (SNRs; Zekveld et al. 2011b, 2012, 2013). The use of a visual text cue ensured that the quality of the cue remained constant while the auditory presentation of the sentence was degraded (cf. Jones & Freyman 2012). Furthermore, we compared cues that were either semantically related or unrelated to the sentences in order to gain insight into how the congruency of information provided by the preceding text cues might influence the processes underlying the effect of contextual information on speech understanding (Goy et al. 2013). In line with previous studies (e.g., Zekveld et al. 2011b; Wild et al. 2012) investigating the effect of text cues on the understanding of degraded speech, we observed that the benefit in performance from word cues that were semantically related to the sentences was generally larger in more adverse listening conditions (Zekveld et al. 2011b). Relative to sentence repetition for unrelated or nonword cues (unpronounceable text strings, such as “rtpa”), when semantically related word cues were presented, sentence repetition accuracy increased from around 25% correct to around 40% correct, depending on the SNR condition (Zekveld et al. 2011b, 2012). Although sentence understanding was aided by semantically related information, the lack of significant difference between the results for unrelated and nonword cues suggests that participants did not base their responses solely on the visual cues insofar as sentence understanding was not hampered by the unrelated cue words (Zekveld et al. 2011b).

### Cognitive Processing and Effort Across SNR Conditions

In the current study, we aimed to examine if the semantic relationship between preceding text cues and sentences influenced the effort expended during a sentence repetition task. Effort is defined as “the deliberate allocation of mental [cognitive] resources to overcome obstacles in goal pursuit when carrying out a [listening] task” (Pichora-Fuller et al. 2016. In general, the effort allocated to listening across a broad range of intelligibility levels follows an inverted U-shaped curve (Zekveld & Kramer 2014; Ohlenforst et al. 2017). At very high intelligibility levels, listening effort is low because the task is easy and the demand for cognitive resources is low. At very low intelligibility levels, listening effort can also be low if participants realize that they cannot muster the resources to meet the demands of the task and so give up listening. In contrast, at intermediate intelligibility levels, effort may be highest because participants feel that they have sufficient resources to meet task demands and that it is worth trying to expend effort to understand speech.

Importantly, both listening effort and the facilitative effects of supportive semantic cues could vary with the uncued intelligibility of the speech across different SNR conditions. In lower SNR conditions, the presentation of cues may facilitate sentence understanding, but effort may also increase if listeners persist in trying to understand the sentences rather than giving up. In higher SNR conditions, the effect of cues on intelligibility is smaller and effort may also be reduced because it is easier to understand speech. Wild et al. (2012) assessed the influence of text cues on the ability of listeners to process spoken sentences that were clear or severely degraded. Listeners were asked to rate the amount of noise they heard in each sentence. They observed that, relative to nonword cues, cues that were semantically related to the sentences were associated with lower subjective noise ratings for degraded speech. This finding was replicated by Signoret et al. (2017) who also demonstrated that additional subjective benefit accrues from semantic coherence and showed that these predictive effects are related to individual differences in working memory capacity. In the study by Wild et al., brain activation was larger for semantically related cues relative to unrelated cues, but only when degraded speech was presented. The influence of textual information on activation in primary auditory cortex supports the notion that the brain actively predicts perceptual input based on prior knowledge (Beck & Kastner 2009; Blank & Davis 2016). Furthermore, when the auditory signal was degraded, higher-level brain regions involved in speech processing, such as the superior temporal sulcus and inferior frontal gyrus, were more active for related when compared with unrelated cues. The frontal activation may reflect more effortful listening when related cues are presented in such relatively difficult conditions (Wild et al. 2012; Peelle 2018).

One established measure that is believed to index cognitive processing load during listening is the task-evoked pupil dilation response (Beatty 1982; Kramer et al. 1997; Piquado et al. 2010; Zekveld et al. 2010). The response is regulated by the combined activity of the sympathetic and parasympathetic branches of the autonomic nervous system (Lowenstein & Loewenfeld 1962). It corresponds to the extent of cortical activation during cognitive processing (Siegle et al. 2003). The task-evoked pupillary responses are relatively robust (e.g., independent of baseline pupil diameter, even when responses are small; Beatty & Lucero-Wagoner 2000). Speech intelligibility strongly affects the pupil dilation response; the response is larger for intermediate intelligibility levels when compared with high intelligibility levels, regardless of masker type (Kuchinsky, et al. 2013; Zekveld & Kramer 2014). Also, the pupil dilation response is larger for speech maskers when compared with noise maskers (Koelewijn et al. 2012) and for syntactically more complex sentences when compared with syntactically simple sentences (Piquado et al. 2010). The pupil dilation response has been shown to be larger for listeners with better cognitive abilities (Zekveld et al. 2011a; Koelewijn et al. 2012). This may reflect greater brain activation during listening, consistent with the notion of the pupil response as an index of summative brain activity (Siegle et al. 2003; Zekveld et al. 2014a). Notably, in a study by Winn (2016), the pupil dilation response during listening to sentence-final words that were predicted by the sentence context was smaller when compared with that during the processing of sentence-final words that were not predicted by the sentence context. However, in the study by Winn, intelligibility was relatively high even for low-context sentences (around 60% correct). The main aim of the present study was to examine whether the pupil dilation response would differ between sentences that were semantically related or unrelated to preceding visual text cues and whether this response would depend on the SNR condition.

### Recall

In one of our previous studies (Zekveld et al. 2013), delayed recall of correctly repeated sentences was better when the sentences were preceded by related as opposed to semantically unrelated cues. These findings suggest that semantically related cues not only facilitate sentence understanding but also memory encoding. In the present study, we tested the influence of the semantic relatedness between the cue words and sentences on the ability to recall the cues after sentence processing. Additionally, we examined the pupil dilation response during recall.

Recall is known to be negatively affected when a person articulates irrelevant sounds during maintenance. Therefore, we also tested whether recall of the cue words would be influenced by overt sentence repetition between encoding and recall of the cue words and whether any such effect would be modulated by the relatedness of cue words and sentences. The “phonological loop” concept described by Baddeley (1983, but see also Baddeley 2012) explains this “articulatory suppression” effect. The phonological loop comprises a phonological store that is coupled with an articulatory rehearsal process to enable the temporary maintenance of phonological representations. Articulation of irrelevant sounds suppresses rehearsal of the to-be-remembered items, thereby interfering with the maintenance of items in memory (Baddeley 2012). To our knowledge, it has yet to be established how recall may be affected by the semantic relationship between the to-be-remembered and articulated items. Therefore, in the present study, we measured the effect of sentence repetition on recall accuracy and the pupil dilation response during the recall of the cue words. Participants listened to masked sentences that were preceded by a set of four visually presented cue words whose meaning was either related or unrelated to the spoken sentence. We asked participants to either speak the cue words after listening to each auditory sentence or to repeat the sentence. Participants were subsequently prompted to recall the cue words. Speaking the cue words aloud before and later recalling them was a form of rehearsal of the items in the phonological loop that was expected to improve recall, whereas repeating the sentence before recalling the cue words was a form of articulatory suppression that was expected to reduce recall performance.

### Predictions

The main aim of the present study was to assess the influence of semantically related or unrelated cue words on the pupil dilation response in young listeners during listening to sentences. In line with the results reported by Winn (2016), the pupil dilation response would be expected to be smaller for semantically related sentences compared with unrelated sentences, especially at high intelligibility levels. However, in line with the notion that the pupil dilation response and brain activation are greater when more information is being actively processed by the brain (Wild et al. 2012; Koelewijn et al. 2014a; Lee et al. 2016), the pupil dilation response would be expected to be larger for semantically related sentences compared with unrelated sentences, especially in adverse SNR conditions in which the intelligibility of the uncued sentences is very low. Hence, we expected an interaction effect between SNR and semantic context on the pupil dilation response during listening. Specifically, we predicted that relatively higher effort levels would be reflected in larger pupil dilation when cue words were related compared with when they were unrelated to the sentences, but only in adverse SNR conditions. Furthermore, previous studies unambiguously demonstrated a robust effect of signal degradation (intelligibility) on the pupil dilation response (Zekveld et al. 2010; Koelewijn et al. 2012; Kuchinsky et al. 2013; Ohlenforst et al. 2017), with larger pupil responses for more challenging SNR levels. Therefore, we expected a general effect of SNR on the pupil dilation response, with larger pupil dilation in more adverse conditions. We also assessed whether repeating sentences and the semantic relationship between the sentences and the cue words affected the accuracy of cue recall and also the pupil dilation response during recall. Anticipating an effect of articulatory suppression, we predicted that repeating sentences would adversely affect the recall of the cue words. In case of related sentences, when the general meaning of the combination of four cue words is congruent with the meaning of the sentences, we predicted that this congruency would facilitate the participants’ ability to maintain the cue words in memory and reduce pupil dilation response during recall. A summary of our hypotheses can be found in Table 1.

**TABLE 1.**
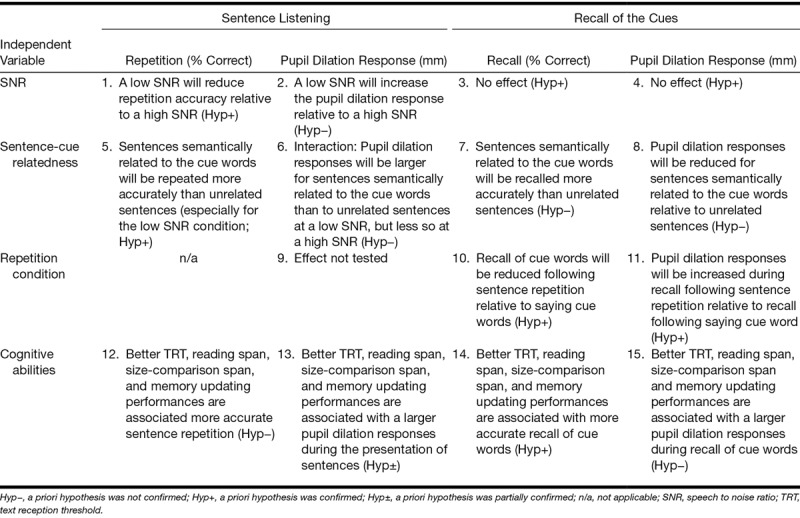
Hypotheses regarding the effects of Sentence-cue relatedness (related, unrelated), SNR (low, high), and Repetition (cues, sentences) on (1) sentence repetition accuracy, (2) the pupil dilation response listening to the presentation of the sentence, (3) the recall of the cues, and (4) the pupil dilation response during recall of the cues

### Individual Differences in Cognitive Abilities

We assessed the possible associations between four cognitive measures that were used to characterize the abilities of the participants and four experimental outcome measures (accuracy of sentence repetition, accuracy of recall of the cue words, and pupil dilation during sentence listening and during the recall of cue words). The cognitive measures included three memory tests and the text reception threshold (TRT) test (Zekveld et al. 2007). Previous research has shown that these abilities are associated with the perceived clarity (Signoret et al. 2017) and accuracy of understanding of cued degraded spoken sentences (Zekveld et al. 2012, 2013). This observed association was consistent with the “resource hypothesis” (Ahern & Beatty 1979; van der Meer et al. 2010; Zekveld et al. 2011a; Koelewijn et al. 2012). According to this hypothesis, increasing the allocation of cognitive resources in response to increased cognitive demand can result in improved performance on speech understanding tasks, particularly in challenging perceptual conditions. In these conditions, listeners may rely heavily on top-down cognitive processing to compensate by using contextual knowledge to resolve ambiguities. The association of better cognitive functioning with an individual’s ability to expend more effort in response to higher task demands has been interpreted as reflecting that more intense use of the brain (i.e., deployment of more cognitive resources) may correspond to increased effort (Zekveld et al. 2011a; Koelewijn et al. 2012; Eckert et al. 2016; Pichora-Fuller et al. 2016). Therefore, we expected that, compared with participants with smaller working memory capacity, those with larger working memory capacity and better ability to read masked text would more accurately repeat sentences, recall cue words, and produce larger pupil dilation responses during listening and recall (e.g., Zekveld et al. 2011a; Koelewijn et al. 2012, 2014b; Zekveld & Kramer 2014).

## MATERIALS AND METHODS

### Participants

Twenty-six normal-hearing young adults (22 women and 4 men; mean age, 22 years; SD = 3 years) participated in the study. They were recruited among students of the VU University and via flyers posted around the university campus. All had Dutch as native language and normal or corrected-to-normal vision as screened with a near-vision screening chart (Bailey & Lovie 1980). Their color vision was screened with Ishihara plates (Ishihara 2001) and classified as normal. The exclusion criteria were the following: pure-tone hearing threshold(s) exceeding 20 dB HL at the octave frequencies between 500 and 8000 Hz, dyslexia or other reading problems, or a self-reported history of a neurological or psychiatric disease. The mean pure-tone hearing threshold of the participants (averaged over both ears and over 0.5, 1, 2, and 4 kHz) was 5.1 dB HL (SD = 4.9 dB HL). All participants provided written informed consent in accordance with the Ethics Committee of the VU University Medical Center.

### General Procedure

The test session started with pure-tone audiometry, near-vision screening, and screening for color blindness. Then, the preparations for pupillometry measurement were conducted (see below), followed by the experimental task (for details, see Experimental Task). After the experimental task, participants performed four cognitive tests (reading span, memory updating, size-comparison span, and TRT). The entire test session took about 2 hours, with a short break halfway.

### Experimental Task

#### Stimuli: Text Cues

**Fig. 1.**
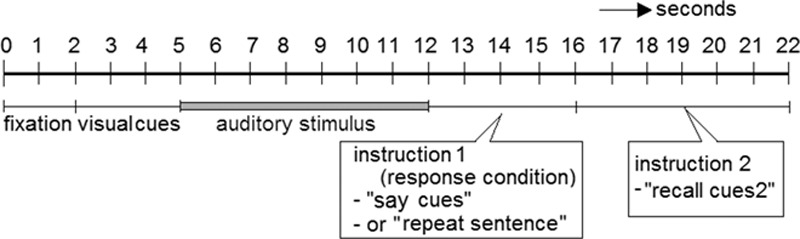
Timing of each trial in the experimental task, for an average sentence length of 1.84 sec. The auditory stimulus consisted of the presentation of the speech masker for 3 sec, followed by the masked sentence (duration between 1.31 and 2.65 sec) and the presentation of the speech masker for 2 sec after the end of the sentence. Participants focused on a fixation dot displayed in the center of the screen during auditory stimulus presentation.

Figure 1 illustrates the timing of events during each trial. At the start of each trial, participants fixated on four strings of hash characters (#) equal to the length of the four text cues presented subsequently. The color of the hash decks and letter characters was gray (presented on a black background), and the Arial font size was 36. The hash decks were presented in the middle of a 22-in screen. The cue words were generated by 27 participants in a previous study (Zekveld et al. 2011b). In that study, we visually presented a selection of sentences (Versfeld et al. 2000) and participants were asked to generate three new words that meaningfully summarized the topic of each sentence (Mäntylä & Nilsson 1988). For example, for the sentence “The child was born last night,” new words could be “baby-delivery-mother.” We counted how often each individual new word was generated across the participants in the study by Zekveld et al. (2011b). In the present study, we presented four words instead of three to increase the amount of semantic context provided. Therefore, for the four most frequently generated words per sentence (as summed over participants), we calculated the sum of these generation frequencies. Finally, we selected the 240 sentences for which this sum was highest. Hence, the four-word cues presented in the current study were new combinations of words that were most often generated by the participants in Zekveld et al.. A similar approach was adopted in Zekveld et al. (2012). The rationale for using orthographic presentations for the cue words was that this ensured correct encoding of the cue words by the participants. Our previous studies demonstrated that text cues are able to substantially enhance the understanding of sentences presented auditorily with masking (Zekveld et al. 2011b, 2012, 2013).

#### Stimuli: Auditory Sentences and Masking

After the presentation of the four cue words, a fixation dot appeared in the center of the screen and participants heard the speech masker for 3 sec. Then, the target sentence started in the continuing speech masker (sentence duration: mean = 1.84 sec, min = 1.31 sec, and max = 2.65 sec) after which there were 2 additional seconds in which only the speech masker was presented. The target speech consisted of sentences spoken by a female speaker (Versfeld et al. 2000). The masker consisted of different sentences from the same stimulus set (Versfeld et al. 2000) that were spoken by a male speaker and were played one sentence at a time without pauses between sentences. The masking signal was spectrally shaped in order to obtain the same long-term average frequency spectrum as the target female speech, but the masker was still clearly recognizable as a male speaking a sentence. For each trial, the audio file containing the masker was started at a random time point in that file.

#### Experimental Test Conditions

Test administration took place in a sound-attenuated room. There were eight experimental speech perception conditions, each consisting of 20 trials. Each trial started with the visual presentation of four-word cues, followed by the auditory presentation of a sentence that was spoken by a female speaker and masked by sentences spoken by a male speaker. Auditory stimuli in the experimental tests were presented by an external soundcard (Creative Sound Blaster Audigy) through Sony MDR V900 headphones (Sony Corporation). A 2 × 2 × 2 within-subjects factorial design was used, with the following factors: Sentence-cue relatedness (sentence related or unrelated to preceding cue words), SNR (low, high), and Response condition (sentence repeated or word cues spoken). The sentences were related to the cue words or not (i.e., for the unrelated condition, the set of four cue words preceding the sentence were related to another sentence that was never presented). The SNR of the sentences was either −17 dB (low SNR) or −10 dB (high SNR). The overall intensity level of the mix of target and masker speech was fixed at 65 dB SPL. The intensity level of the speech was 47.9 dB SPL in the −17 dB SNR condition and 54.6 dB SPL in the −10 dB SNR condition. Immediately after auditory stimulus offset, subjects were asked to either say the sentence they had just heard (sentence repetition) or they were asked to say the four cue words they had read before hearing the sentence. After speaking either the sentence or the cue words, participants recalled the four cue words that had preceded the sentence.

#### Response Conditions

Immediately after auditory presentation of the sentence, the fixation dot disappeared and the first instruction was visually presented: the text “herhaal woorden” (say cue words), or “herhaal de zin” (say sentence), respectively, prompted the participant to say either the cue words or the sentence. Thus, participants were required to respond by speaking on all trials. After 4 sec, a second instruction was presented visually for 6 sec, which was always “herhaal woorden2” (repeat cues2). We added the “2” to the display to ensure that the participants were able to distinguish the second instruction from the first instruction that sometimes prompted the participant to say the cue words so that they would be aware that on these trials they should say the cue words a second time for the recall phase of the trial. Depending on whether participants responded by saying the sentence or the four cue words on a given trial, the experimenter scored either how many words from the sentences or how many cue words had been spoken correctly in the second interval. In order to facilitate the scoring by the experimenter, for each interval, each spoken word in the sentence or each text cue word was presented to the experimenter visually on a button in the software interface. The experimenter used these buttons to indicate the correctness of the response (by means of mouse clicks) and added any additional spoken responses by typing these in a comment field. Sentences were only scored as correct if all of the words in the sentence were correctly repeated.

The order of presentation of the trials from the eight conditions was randomized, with the restriction that trials from the same condition were presented no more than three times in a row, and such that the progression of the eight conditions was equally distributed over the experimental test blocks.

#### Pupillometry

At the start of the experimental speech test, the pupil size was measured in maximum illumination (100 lux) and in complete darkness. The room illumination was adapted individually such that the pupil size was around the middle of its dynamic range at the start of the test. This adjustment of the room illumination prevents ceiling and floor effects in the pupil dilation response and makes the response independent of the baseline pupil size (Beatty & Lucero-Wagoner 2000). The mean room illumination after individual adjustments was 52 lux (SD = 23 lux).

The location and size of the pupil of the left eye were measured during each trial. The pupil response is similar in both eyes (Purves et al. 2004); the analysis of the left eye was an arbitrary choice. We analyzed the pupil size in two intervals in each trial: once during sentence presentation and once during cue recall. The first “sentence presentation” interval comprised the pupil dilation response during listening to the auditory presentation of the masker and sentence. This interval started 1 sec before sentence onset (i.e., during the presentation of the auditory masker) and ended at masker offset for the shortest sentence presented in the set (i.e., 3.3 sec after target speech onset). As a result, for all trials, the interval only contained pupil data registered while participants listened to masked speech. The second “recall cue words” interval comprised the recall of the cues (i.e., the “instruction 2” interval as illustrated in Figure 1). This interval during which pupil dilation was measured started 1 sec before the second recall instruction appeared on the screen and ended 7 sec later.

Pupil diameters below 3 SDs of the mean diameter during each trial were coded as a blink. If the data contained more than 15% blinks in the interval of interest, the data for this interval were excluded from data analysis. Furthermore, the pupil data were corrected for possible artifacts due to eye movements. We visually inspected the plotted eye movements and omitted trials in which there were numerous eye movements that substantially deviated from the center of the screen. On average, the pupil data for 17 of the 20 trials in each condition were included for the sentence presentation interval, and pupil data from 16 of the 20 trials per condition were included for the recall cue words interval. Eye blinks were replaced by linear interpolation starting four samples before and ending eight samples after a blink. The data were passed through a five-point moving average smoothing filter. The data were then averaged over trials for each of the condition, separately for the sentence presentation and for recall cue words intervals. Then, the mean pupil diameters in the first second of the sentence presentation interval and in the first second of the recall cue words interval were defined as baseline diameter 1 (for sentence presentation) and baseline diameter 2 (for recall), respectively. We subsequently determined the peak pupil dilation (peak dilation amplitude in mm) in both intervals relative to baseline diameter 1 (for sentence presentation) or baseline diameter 2 (for recall of the cues). We also report the mean pupil dilation (average pupil dilation relative to the baseline pupil size in the interval of interest). Usually, the peak and mean pupil dilation are relatively strongly associated with each other. Therefore, we expected similar patterns of results for the two parameters. The mean pupil dilation data are reported in order to provide a more complete overview of the results.

#### Reading Span Test

The reading span test assesses complex verbal working memory capacity (Daneman & Carpenter 1980). The Dutch sentence material and test were developed to be equivalent to the Swedish version described by Andersson et al. (2001), see Besser et al. (2012). In the reading span test, five-word sentences were presented visually as text. Half of the sentences were nonsense sentences (e.g., the table sings a song); the other half made sense (e.g., the friend told a story). First, three sets of three sentences were presented, followed by three sets of four sentences, three sets of five sentences, and three sets of six sentences. Immediately after each sentence, participants verbally indicated whether the sentence made sense or not. After each set of sentences, participants were prompted to recall, in serial order, either the first or the last word of each sentence in the set. The experimenter recorded the number of words correctly recalled, regardless of order.

#### Size Comparison Span

The size-comparison span task (Sörqvist et al. 2010) measures verbal working memory capacity and the ability to suppress irrelevant information. Sets of size-comparison questions like “is a BUSH larger than a TREE?” were presented visually. Immediately after each question, participants responded to the question by pressing one of two buttons corresponding to “yes” or “no.” The response prompted the disappearance of the question and the presentation of a semantically related and to-be-remembered word like FLOWER was presented. Ten sets of question word pairs were presented in total; the five set sizes ranged from two to six question pairs with each set size being presented twice. Within sets, nouns used in the questions and those to be remembered were from the same semantic category, but between sets these categories differed. After each set, participants were asked to orally recall the to-be-remembered words. The score was the total number of correctly recalled words regardless of the order in which they were recalled (maximum of 40). Higher scores reflected better working memory capacity. As the participant has to inhibit the semantically related sentence words while recalling the to-be-remembered words, this test is assumed to tap into both working memory processing and inhibition ability (Sörqvist et al. 2010). Both abilities may facilitate the speech understanding in challenging listening conditions.

#### Memory Updating

In the memory updating test (Morris & Jones 1990; Miyake et al. 2000), sequences of consonants are presented visually. Participants are asked to keep the four most recently presented consonants in memory. The DMDX platform (Forster & Forster 2003) was used to present 12 lists of 5, 7, 9, or 11 consonants serially as text at the center of the computer screen. The lists with different lengths were presented in random order. No consonant appeared twice in the same list. Two lists consisting of seven and nine letters were presented as practice. Participants were asked to repeat the last four consonants when prompted at the end of the list. The score was the total number of consonants correctly recalled irrespective of order. A high score on this test indicates a good ability to maintain information in working memory while at the same time keeping it continually updated. This ability is likely to be called on during speech understanding in noise to determine when undisambiguated fragments of speech should be discarded in favor of new information which may lead to more complete understanding.

#### TRT Test

The TRT test (Zekveld et al. 2007) is a text-based test that measures the reader’s ability to understand degraded verbal information. Thirteen partly masked printed sentences (Versfeld et al. 2000) were presented. The sentences were different to those presented in the experimental test. The text was masked with a bar pattern (see Zekveld et al. 2007). The field background color was white, text color was red, and the color of the mask was black. At the start of each trial, the mask became visible and the sentence appeared as text “behind” it in a word-by-word fashion. The percentage of unmasked text at the start of the test was relatively low (difficult). The display onset of each word in the sentence was timed to the start of the word in the corresponding audio file (Versfeld et al. 2000). All words remained on the screen for 3.5 sec after the sentence was complete. Participants were asked to read the sentences aloud. The experimenter scored whether the sentences were reproduced entirely correctly. A one-up, one-down adaptive procedure with a step size of 6% of masking was used to determine the percentage of unmasked text required for the participant to read 50% of the sentences entirely correctly. The test was performed three times; the data from the first practice test were omitted from the analysis. The TRT was calculated as the mean percentage of unmasked text on the remaining two tests, with lower TRTs indicating better performance.

### Setup

The audiogram was made using an audiometer (Decos Systems B.V., software version 2010.2.6) connected to TDH 39 headphones. Subjects were seated behind an SMI iView X RED remote eye-tracking system with spatial resolution of 0.03° and sampling frequency of 60 Hz. A PC screen was positioned on top of the pupillometric system, about 45 cm away from the subject’s head.

### Statistical Analyses

First, descriptive statistics of the behavioral measures were calculated. We performed a repeated measures analysis of variance (ANOVA) with within-subject factors SNR (low, high) and Sentence-cue relatedness (related, unrelated) on sentence recognition (Hypotheses [Hyp] 1 and 5, see Table 1). We furthermore performed a repeated measures ANOVA on cue-recall performance with the same factors as described above and a third within-subject factor Response condition (sentences or cue words; Hyp 3, 7, and 10). We performed a post hoc analysis of the errors in the recall task. Finally, two-factor and three-factor repeated measures ANOVAs similar to those performed on the behavioral data were performed on the pupillometry data (peak and mean pupil dilation) obtained during presentation (Hyp 2 and 6) and recall of cue words (Hyp 4, 8, and 11). We expected similar results for the peak and mean pupil dilation data (e.g., Zekveld et al. 2010, 2011a).

We performed four backward regression analyses (prediction models) to assess to what extent the variance in sentence repetition accuracy, cue word recall accuracy, and the peak pupil dilation during the sentence presentation and recall phases of the trials was predicted by the four cognitive tests, TRT, reading span, size-comparison span, and memory updating performance (Hyp 12, 13, 14, and 15). For these analyses, we averaged the accuracy or peak pupil dilation across all conditions in order to limit the number of analyses. For the same reason, we only performed these analyses on the peak pupil dilation data, and not on the mean pupil dilation data. Multicollinearity was assessed by calculating Pearson correlation coefficients between the potential predictors and confounders and by assessing the variance inflation factor (VIF, Cohen et al. 2013) of the starting model. A VIF < 5 criterion was applied, and all VIFs were below 1.5, indicating no multicollinearity. A variable was excluded from the model if the *p* value of the relationship with the dependent variable was >0.10. This exclusion criterion for individual predictors was used to increase the predictive power of the regression model (Sauerbrei 1999; Steyerberg 2008).

## RESULTS

### Behavioral Data

**Fig. 2.**
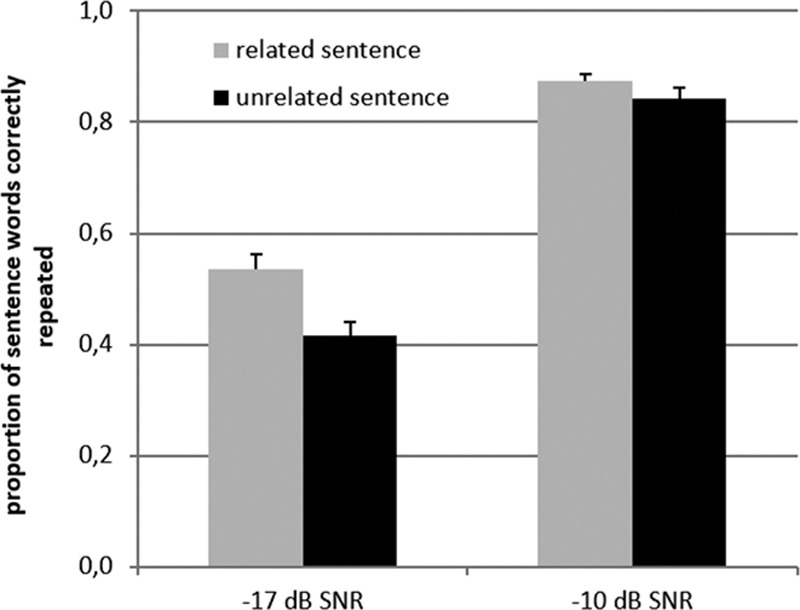
Proportion of sentence words correctly repeated in the sentence-repetition conditions, shown separately for the two signal to noise ratios (SNRs) and for the conditions in which the cue words were related to the sentences and the conditions in which the cue words were unrelated to the sentences. Error bars indicate ±1 SE.

Figure 2 shows the means for the proportion of sentence words that were correctly repeated in each of the four sentence-response conditions. The main effects of Sentence-cue relatedness (*F*_(1,25)_ = 36.9, *p* < 0.001, partial η^2^ = 0.60) and SNR (*F*_(1,25)_ = 439, *p* < 0.001, partial η^2^ = 0.95) were statistically significant. As expected, the proportion of sentence words correctly repeated after the presentation of the sentence was greater for the higher when compared with the lower SNR, and it was also greater for sentences that were preceded by semantically related cues when compared with unrelated cues. The ANOVA also indicated an interaction effect between SNR and Sentence-cue relatedness (*F*_(1,25)_ = 12.9, *p* = 0.001, partial η^2^ = 0.34). As shown in Figure 2, this interaction is based on a larger effect of Sentence-cue relatedness for the lower SNR of −17 dB. Post hoc paired-samples *t*-tests confirmed that the effect of Sentence-cue relatedness was only statistically significant for the −17 dB SNR level (*t*_*(25*)_ = 6.3, Bonferroni-corrected *p* < 0.01), and not for the −10 dB SNR level (Bonferroni-corrected *p* > 0.10). Note that one of the disadvantages of using proportional scores is that the scale values are not linear in relation to test variability (Studebaker 1985). The same analyses were performed on the rationalized arcsine-transformed (Studebaker 1985) scores in order to assess whether the results were influenced by the distribution of the data and the same pattern of results was found.

**Fig. 3.**
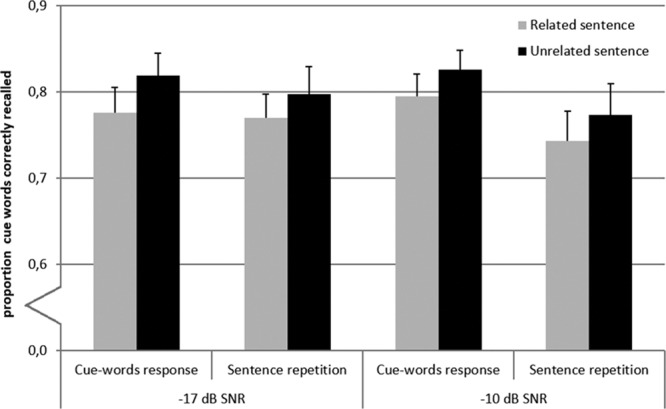
Proportion of cues correctly recalled for each of the eight conditions (2 signal to noise ratios [SNRs] × 2 sentence-cue relatedness conditions [related vs. unrelated] × 2 response conditions [sentences vs. cue words]). Error bars indicate ±1 SE.

Figure 3 shows the descriptive statistics for the accuracy of recall of the presentence text word cues. An ANOVA with factors Sentence-cue relatedness, SNR, and Response condition (say sentence that was heard or cue words that were read) showed a main effect of Sentence-cue relatedness (*F*_(1,25)_ = 12.9, *p* = 0.001, partial η^2^ = 0.34) and a main effect of Response condition (*F*_(1,25)_ = 5.9, *p* = 0.02, partial η^2^ = 0.19). There was no statistically significant effect of SNR. As shown in Figure 3, recall of the cues was poorer when they were related compared with when they were unrelated to the sentence, and also when participants were asked to say the sentences instead of the cues before the recall cue words interval. The interaction effects between the factors were not statistically significant (all *p* values > 0.17). The analysis using rationalized arcsine-transformed (Studebaker 1985) proportions yielded the same results.

We expected that the recall of the cues would be better when the sentences were related to the cues. One factor potentially contributing to the opposite effect that was observed is possible confusion between the cues and the sentence words. Such confusion could be reflected by the erroneous recall of sentence words instead of the cues during the recall phase. To examine this possibility, we performed a post hoc error analysis. We counted the number of intrusion errors that consisted of sentence words or morphological variants of sentence words. The total number of intrusion errors is presented in Table 2 for each condition. As sentence repetition accuracy was poorer at lower SNRs (see Fig. 2), the number of intrusion errors should be lower in the −17 dB SNR conditions when compared with the −10 dB SNR conditions. This was assessed as well. Finally, we examined whether the total number of errors (summed over trials per condition) differed depending on whether the cues words were related or unrelated to the sentences (see Table 2.).

**TABLE 2.**
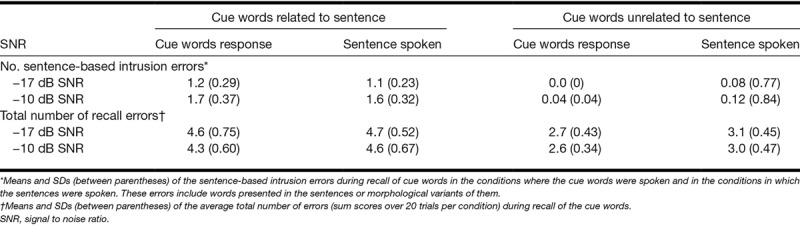
Sentence-based intrusion errors during recall of cue words in the conditions where the cue words were spoken and in the conditions in which the sentences were spoken

As the distribution of the error frequencies was highly positively skewed, we used Wilcoxon signed ranks tests in the error analyses. A Wilcoxon signed ranks test of the main effect of Sentence-cue relatedness indicated that the number of sentence-related intrusion errors was significantly larger when the sentences were related to the cues compared with when they were unrelated (*z* = −3.9, *p* < 0.001). A second test assessing the main effect of SNR indicated that the number of intrusion errors was significantly larger in the less adverse (−10 dB SNR) when compared with the more adverse (−17 dB SNR) conditions (*z* = −2.3, *p* < 0.05). Finally, we observed a main effect of Sentence-cue relatedness on the total number of errors which was larger when the cues were related to the sentences compared with when they were unrelated (Wilcoxon signed ranks test *z* = −3.9, *p* < 0.001). These results imply that participants made more sentence-based intrusion errors when the cues to be remembered were related to the sentences, and for sentences presented at −10 versus −17 dB SNR, and that those errors were made in addition to the errors that were not words that had been heard in sentences.

### Pupillometry Data

**Fig. 4.**
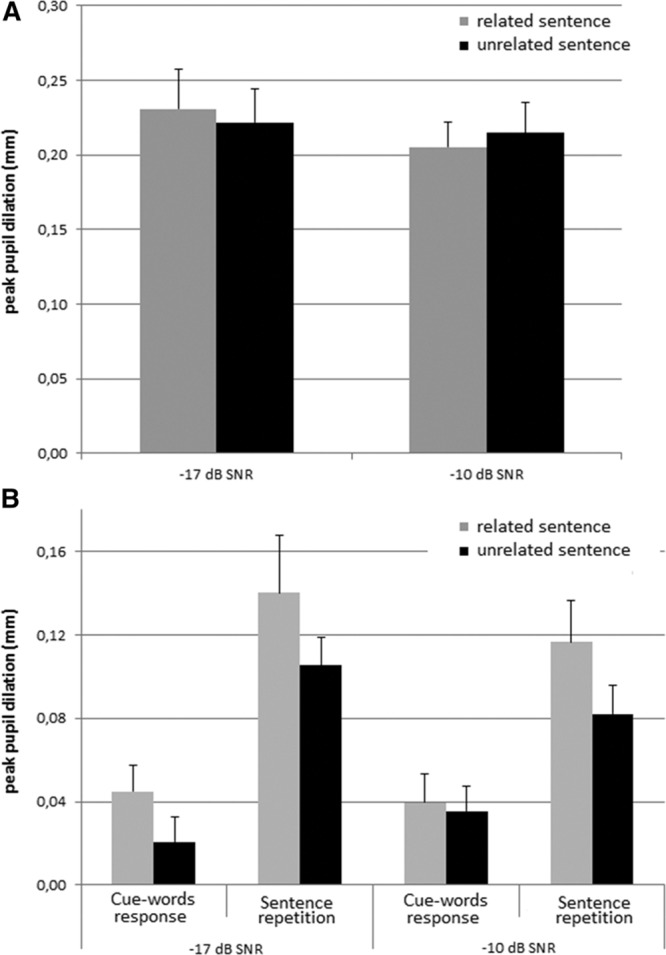
Pupil dilation results. A, Peak pupil dilation relative to baseline1 (between 7 and 8 sec, see also Fig. 1 for a depiction the timing of the trials), for the two SNRs and for semantically related and unrelated sentences. B, Peak pupil dilation relative to baseline2 (see Fig. 1) during recall of the cue words in each of the eight conditions (2 signal to noise ratios [SNRs] × 2 sentence-cue relatedness conditions [related vs. unrelated] × 2 response conditions [sentences vs. cue words]).

Figure 4A shows the mean peak pupil dilation during the interval when the auditory sentence was presented. Note that the ANOVA with factors Sentence-cue relatedness and SNR showed no statistically significant effects of these two factors on the peak pupil dilation and no interaction effect (all *p* values > 0.15). In contrast to previous findings, the peak pupil dilation during listening to masked speech does not seem to be affected by differences in SNR and the resulting intelligibility differences in the conditions. The mean pupil dilation during listening is presented in Table 3. An ANOVA showed no statistically significant effects of Sentence-cue relatedness and SNR on the mean pupil dilation and no interaction effect (all *p* values > 0.24).

**TABLE 3.**
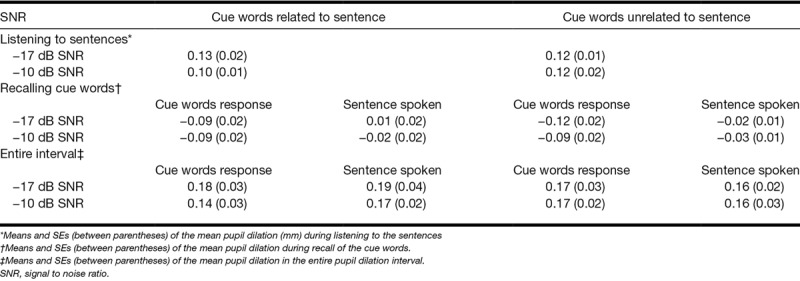
Means and SEs (between parentheses) of the mean pupil dilation (mm)

We additionally performed an ANOVA with factors Sentence-cue relatedness, SNR, and Response condition on the peak pupil dilation (Fig. 4B) in the final (second) recall interval. The results indicated a main effect of Sentence-cue relatedness (*F*_(1,25)_ = 4.6, *p* = 0.04, partial η^2^ = 0.16) and a main effect of Response condition (*F*_(1,25)_ = 27.1, *p* < 0.001, partial η^2^ = 0.52) on the peak pupil dilation. As expected, the peak pupil dilation was smaller when participants recalled the cues after having said them. Unexpectedly, it was also smaller when the cue words were semantically unrelated to, rather than related to, the sentences. An ANOVA assessing the effect of these factors on the mean pupil dilation (see Table 3) indicated effects of Sentence-cue relatedness (*F*_(1,25)_ = 6.8, *p* = 0.015, partial η^2^ = 0.21) and Response condition (*F*_(1,25)_ = 23.6, *p* < 0.001, partial η^2^ = 0.49). In addition, an interaction effect between SNR and Response condition was observed (*F*_(1,25)_ = 4.3, *p* = 0.05, partial η^2^ = 0.15, with more pronounced effects of Response condition for the −17 dB SNR than the −10 dB SNR conditions (*t*_*(25*)_ = −2.1, *p* < 0.05).

**Fig. 5.**
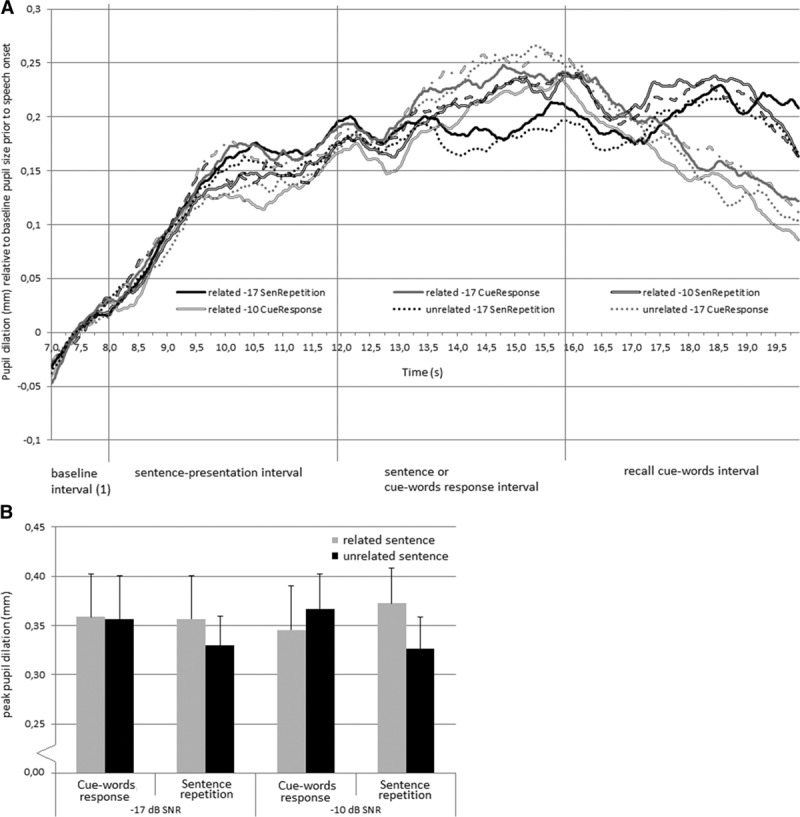
Pupil dilation response in entire trial interval. A, Pupil dilation response relative to the pupil size in the baseline interval prior speech onset (i.e., baseline1 between 7 and 8 sec, see also Fig. 1). The vertical lines indicate the intervals of interest for an average sentence length of 1.84 sec. B, Maximum peak pupil dilation in the entire interval between speech onset (8 sec after trial onset) and the end of the trial. Note that the values do not directly correspond to the data illustrated in A because the latency of the peak pupil dilation for each condition differed between individuals.

The counterintuitive effect of Sentence-cue relatedness on the peak pupil dilation during recall of the cues may have been influenced by differences between the conditions in the time required by the pupil to return to baseline following listening to the sentence. Figure 5A shows the pupil dilation between sentence onset and the end of the trial relative to the average pupil size in baseline interval 1 (before onset of the sentence). Note that the two intervals (sentence presentation interval and final recall interval) that contained the pupil dilation data of interest are both included in Figure 5A. However, a separate baseline correction was applied in the analyses of pupil responses for the recall interval. For some of the conditions, the maximum peak pupil dilation of this overall pupil response is located in the interval between sentence presentation and the final recall of the cue words (i.e., outside the a priori determined intervals of interest). Therefore, we decided to add a post hoc analysis of the effects of Sentence-cue relatedness, SNR, and Response condition on the overall maximal peak pupil dilation in the interval between sentence onset and end of the trial, relative to the baseline pupil size before the onset of the sentence (see Fig. 5B). This interval will be referred to as the entire pupil dilation interval. This post hoc ANOVA revealed no significant main effects, but a significant interaction effect between Sentence-cue relatedness and Response condition on the peak pupil dilation in the entire pupil dilation interval (*F*_(1,25)_ = 5.92, *p* = 0.022, partial η^2^ = 0.19). This indicates that the peak pupil dilation was smaller when the cue words were unrelated to the sentences, in particular in the response conditions in which participants spoke the sentences (*t*_*(25*)_ = −2.4, *p* < 0.05). In addition to the peak pupil dilation data, the mean pupil dilation data were analyzed. Table 3 shows the mean pupil dilation in the entire interval. An ANOVA showed no statistically significant main or interaction effects of the three factors on the mean pupil dilation (all *p* values > 0.05).

### Correlation and Regression Analyses

Table 4 presents the descriptive statistics for the results on the cognitive tests, as well as the Pearson correlation coefficients between these cognitive test scores and results on the experimental measures (sentence repetition accuracy, accuracy of recall for the cue words, and the peak pupil dilation during sentence presentation and final recall of the cue words [averaged over conditions]). Table 4 also shows the number of participants who completed each test as a few of the participants were not able to complete all cognitive tests due to logistical problems.

**TABLE 4.**
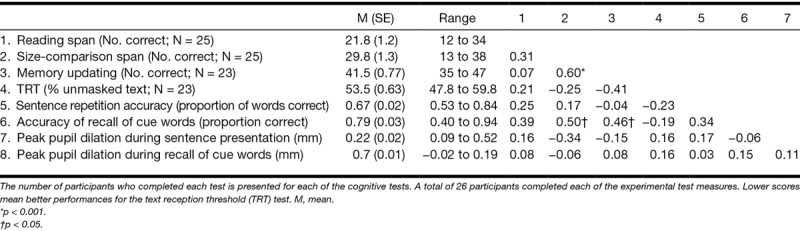
Descriptive statistics and Pearson correlation coefficients between scores on the three cognitive tests and on experimental test measures (accuracy of sentence repetition, accuracy of recall of cue words, and the peak pupil dilation during listening to sentences and during the recall of cue words)

Participants who had a relatively large size-comparison performance also had a relatively good memory updating performance (Pearson *r* = 0.60, *p* < 0.01). Furthermore, better performance on both the size-comparison span and memory updating tests were associated with better recall of the cue words (Pearson *r* = 0.50, *p* < 0.05 and Pearson *r* = 0.46, *p* < 0.05, respectively). The remaining Pearson correlation coefficients were not statistically significant.

No significant prediction models were yielded by the backward regression analyses assessing the predictive value of the reading span test, size-comparison span test, memory updating, and TRT (independent variables) in explaining interindividual variance in the experimental measures of the proportion of sentences correctly repeated or the peak pupil dilation during recall of the cue words. The prediction model of the proportion of correctly recalled cue words is presented in Table 5. Larger reading span and better memory updating were associated with better recall of the cues (*F*_(2,20)_ = 5.4, *p* = 0.013). These factors accounted for 35% of the variance in cue recall. Finally, larger reading span capacity but lower size-comparison span capacity was associated with higher peak pupil dilation during sentence presentation, accounting for 23% of the variance (*F*_(2,20)_ = 3.0, *p* = 0.07).

**TABLE 5.**
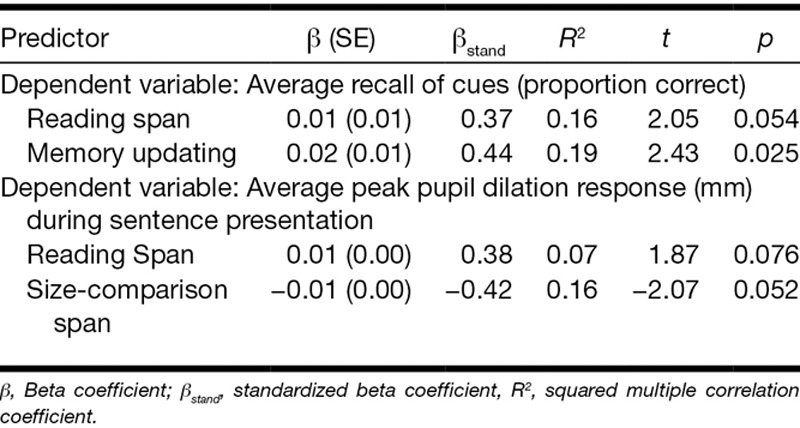
Results of the backward regression analyses models for the average recall of cues and the average peak pupil dilation response

Finally, as described above, the present data did not show evidence for the expected effects of SNR and Sentence-cue relatedness on the pupil dilation response during listening. To explore whether cognitive ability interacted with these effects, we calculated the Pearson correlation coefficients between the four cognitive measures and the average effect of SNR and Sentence-cue relatedness on the peak pupil dilation response (i.e., by means of calculating difference scores between the conditions). These associations were not statistically significant, indicating that ability to make linguistic inferences and working memory capacity likely did not influence these effects.

## DISCUSSION

In the present study, we investigated whether the facilitation of speech understanding in noise achieved by providing preceding semantically related text cues is associated with increased cognitive load. Additionally, we tested the effect of saying cue words or sentences and the effect of the semantic relatedness between the cue words and sentences on the recall of the text cues and on the pupil dilation response during recall. Importantly, in contrast to previous studies, participants were simultaneously engaged in two concurrent tasks: listening to sentences and remembering cue words presented as text before the sentences.

### Effect of SNR

The results of the current study are summarized in Table 1 by the “Hyp−” (hypothesis was not confirmed), “Hyp+” (hypothesis was confirmed), and “Hyp±” (hypothesis was partially confirmed) markings. The main finding of the current study was that the memory load imposed by the recall task nullified the well-established, robust effect of SNR on the pupil dilation response during listening to masked speech. The relatively large (7 dB) SNR difference applied in the current study resulted in large effects on intelligibility (see Fig. 2). The absent effect on the peak and mean pupil dilation response is in sharp contrast to the results of many previous studies conducted in our lab (e.g., Zekveld et al. 2010; Zekveld et al. 2011a; Koelewijn et al. 2012; Zekveld & Kramer 2014; Zekveld et al. 2014a) and elsewhere (Kuchinsky et al. 2013; McGarrigle et al. 2017). For example, in one of these prior studies that used the same setup and stimuli and tested participants from a similar population, there was a relatively large difference in the peak pupil dilation response of 0.1 mm for a difference in intelligibility level comparable to the one observed in the present study (Zekveld & Kramer 2014). However, in the current study, the participants listened to the sentences just after they had read the four cue words and attempted to encode these cue words in memory. It is likely that the participants concentrated on cue maintenance while listening to the sentences, as recall was required in each trial, whereas sentence repetition was only required in half of the trials. This may have influenced the relative priority participants gave to the two tasks and perhaps also the cognitive resources allocated during listening.

The pupil size during the entire course of the trial as presented in Figure 5A indicates that for each of the conditions, the maximum pupil size is reached well after the presentation of the auditory stimulus. The processes reflected by these later peaks cannot be attributed solely to listening to the sentences because in these later time intervals participants were already saying the cue words they had read or repeating the sentences they had heard. Furthermore, no effects of SNR were observed when the overall peak pupil response during the entire course of the trial after sentence onset was analyzed.

The imposed memory load was the same (i.e., recall of four cue words) in each condition, which is in line with the similar pupil dilation response for each of the conditions during and shortly after sentence offset. The current data indicate that the engagement of the participants in the maintenance of the word cues in memory, and concomitant allocation of cognitive resources to memory during listening, can overshadow well-established effects of SNR on the peak and mean pupil dilation during speech understanding. The peak pupil dilation in response to speech understanding tasks can reach levels of around 0.50 mm (Koelewijn et al. 2014a). In the present study, the maximum dilation was around 0.36 mm (see Fig. 5B). Therefore, it is unlikely that the current absence of an effect of SNR on the peak and mean pupil dilation is due to the pupil size approaching its maximum size (e.g., Beatty & Lucero-Wagoner 2000).

### Effect of Semantic Relationship Between Cue Words and Sentences

In the current study, we replicated the relatively large effects of context on the accuracy of sentence repetition (Zekveld et al. 2011b, 2012, 2013). However, the effect of the semantic relationship between the sentences and the cue words on the pupil dilation response during listening was not statistically significant in the present study. This absence of this effect is in line with the results of one of our previous functional magnetic resonance imaging studies that showed no difference in brain activation during listening to sentences that were either related or unrelated to preceding text cues (Zekveld et al. 2013, but see e.g., Obleser & Kotz 2009 for contrasting findings). The maintenance of the cues in memory may have influenced the pupil response during listening to the sentences to an unknown extent. Therefore, it would be interesting to assess the effect of contextual cues on the pupil dilation response when no additional memory load is imposed before listening. The association between larger reading span (working memory capacity) and larger peak pupil dilation during listening confirms previous findings (Zekveld et al. 2011a; Koelewijn et al. 2012, 2014b) and is consistent with the “resource hypothesis” (Ahern & Beatty 1979; van der Meer et al. 2010) that individuals with larger working memory capacity allocate more resources or exert more effort during listening (see also the Framework for Understanding Effortful Listening (FUEL); Pichora-Fuller at al. 2016). In contrast, the current regression analyses furthermore showed that larger size-comparison span was associated with smaller peak pupil dilation during listening. It is unclear why these relatively similar working memory tests had opposite directions of associations with the pupil dilation response during listening. Note, however, that the univariate associations between the two variables on the one hand and the peak pupil dilation during listening on the other hand were not statistically significant. Hence, these associations only became apparent when both variables were included in the prediction model. Both reading span and size-comparison span tests measure working memory capacity, but the latter the ability to suppress irrelevant information (Sörqvist et al. 2012). Speculatively, better ability to suppress irrelevant information may reduce the allocation of listening effort in the complex task currently applied, whereas larger working memory capacity is in itself associated with increased listening effort. More research is needed to further assess these hypotheses.

In contrast to previous studies, better cognitive functioning was not associated with better sentence repetition accuracy across conditions. The absence of this association is probably not due to potential ceiling effects as the average repetition accuracy across conditions was around 65% correct. However, better reading span and memory updating performance were associated with better recall of the cues. This finding is in line with the expected reliance on (working) memory for remembering speech once it has been heard.

The current findings are consistent with our previous work (Zekveld et al. 2014b) in which we reported evidence suggesting that although central (e.g., auditory-stream segregation) and peripheral auditory factors may both influence word recognition accuracy, central auditory factors may be more strongly associated with cognitive processing load than peripheral auditory factors. We observed that the pupil dilation response is sensitive to sentence-cue relatedness during recall of the cue words and associated working memory capacity, but that there was no effect of sentence-cue relatedness or SNR on pupil dilation during sentence presentation. This suggests that the pupil response predominantly reflected the cognitive load associated with the memory task rather than the changes in perceptual load during listening. The implications of this finding are highly relevant for future studies applying pupillometry to assess speech understanding: applying a (external) memory or cognitive load during speech processing seems to completely override the well-known intelligibility effects on the pupil dilation response. The clinical implication of this finding is that cognitive load may be a more important factor to take into account than perceptual load when it comes to listening effort. This finding may relate to recent results of a functional magnetic resonance imaging study by Sörqvist et al. (2016) who showed that increasing the cognitive load in an attended visually presented memory task suppresses the neural response to task-irrelevant auditory stimuli in cortical and subcortical areas. The authors of that study argue that focusing attention on a visual working memory task shields against auditory distraction (Sörqvist et al. 2012, 2016; see also Molloy et al. 2015).

Interestingly, the current study showed that recall of the cues was poorer in trials with cue words that were related when compared with cue words that were unrelated to the sentences. Also, the pupil response during recall indicated that cognitive processing load was larger for recalling cue words that were followed by semantically related sentences when compared with semantically unrelated sentences. This may suggest that the storage of the cues in memory, and the active maintenance of the cues in memory, is disrupted more by sentences that are semantically related to the cues when compared with unrelated sentences. This disruptive effect occurred despite the facilitative effect of related sentences on sentence repetition (Zekveld et al. 2011b, 2012, 2013).

This possibility was supported by a post hoc analysis of the recall errors indicating that if the presentation of the visual cues was followed by a semantically related sentence, participants made more sentence/word-based intrusion errors when compared with the unrelated sentences. Furthermore, there were more intrusions when the sentence s were more intelligible. This may have been because more sentence words were encoded, thus causing greater confusion, when intelligibility was higher.

Similar findings have been observed in studies of reading (Gordon et al. 2001; Ledoux & Gordon 2006; Van Dyke & McElree 2006). In the study of Gordon et al. (2002), participants were asked to remember three words (proper names or roles) before reading a sentence with nouns phrases that were either proper names or roles (other than the to-be-remembered ones). Gordon et al. showed that when the type of to-be-remembered nouns matched to those in the sentences, the participants made more errors on subsequent comprehension questions.

The current data could suggest that both cue words and sentence words are encoded in working memory and that the closer they are semantically, the harder it is to remember whether they were cues or sentence words. In a review by Gallo (2010), an activation/monitoring framework was postulated as a mechanism underlying intrusion errors (often called “false memories,” Deese 1959, Roediger & McDermott 1995) that are similar to the ones observed in the present study. According to this framework, intrusion errors can be the result of source-monitoring problems (Johnson et al. 1993).

In the −17 dB SNR condition, the intelligibility of the speech was lower than in the −10 dB condition (see Fig. 2). This lower SNR could have resulted in a smaller effect of sentence-cue relatedness on the recall of the cue words, as fewer sentence words were intelligible and hence there was less potential semantically interference. However, the effect of Sentence-cue relatedness on number of cues correctly recalled was the same regardless of the SNR at which the sentences were presented. SNR did influence the number of intrusion errors (i.e., erroneously recalling sentence words instead of cue words). This number was smaller for the −17 dB SNR condition than for the −10 dB SNR condition.

### Effect of Response Condition

Another finding of the present study was that both the pupil dilation response during recall of the cues and recall accuracy were influenced by the response condition. Recall accuracy was lower, and the peak and mean pupil dilation were larger when participants first said the sentence instead of recalling the cue words (see Figs. 3 and 4B). For the mean pupil dilation during recall, this effect was larger for the −17 dB SNR than the −10 dB SNR condition. The current data are consistent with working memory models including a phonological loop for rehearsal of to-be-remembered verbal items, which is disrupted more by articulation of heard sentences than by passive listening (Baddeley 2012). We did not record the cue words spoken in the first response interval. However, we noticed instances in which participants correctly recalled cue words that they did not utter during the first response interval, possibly because they had more time to think of possible candidates. However, the reverse pattern also occurred. The higher performance in the conditions in which the participants recalled the cue words after having spoken them could also be related to the “testing effect” (e.g., Carpenter 2009), whereby retrieval attempts increase the retention of information in long-term memory. However, in the present study, the contribution of this effect was likely relatively small because the recall delay was very short and the testing effect is larger when there is a longer time delay (Roediger & Karpicke 2006). In general, the recall of the cue words was better for individuals with larger (working) memory capacity as assessed by the reading span and memory updating tests, possibly because they prioritized maintenance of cue words over competing processing demands (see Hunter & Pisoni 2018).

## CONCLUSIONS

The current study assessed the influence of the semantic relationship between four-word text cues and the accuracy with which participants repeated sentences presented auditorily in noise, the recall of the cue words, and the pupil dilation response during sentence presentation and the recall of the cue words. The findings demonstrate that if speech understanding takes place under a memory load, large differences in SNR remarkably do not affect the pupil dilation response, despite large effects on sentence repetition accuracy. Also, a semantic relationship between the cue words and sentences did facilitate the perception of the sentences, but did not influence the peak pupil dilation during the retention of the cue words for later recall. The findings demonstrate that a concurrent memory task can eliminate established effects of auditory stimulus characteristics on the peak and mean pupil dilation, even though established behavioral effects are preserved, possibly suggesting that the peak and mean pupil dilation response are more sensitive to central factors (memory load) than peripheral factors. In contrast to the facilitation of speech understanding when cue words and sentences were semantically related, the recall of the cue words was reduced in these conditions relative to the unrelated conditions. We argue that semantic distraction or confusability between the sentence words and the cues result in this interference effect. This interference was also reflected by a larger peak and mean pupil dilation response during cue recall relative to the peak and mean pupil dilation response for unrelated sentences. This indicates that recalling the semantically related cues requires more cognitive processing load. Repeating sentences resulted in poorer recall of cue words overall and larger pupil dilation during cue recall, an effect which is likely driven by articulatory interference. Finally, the correlation and linear regression analyses showed that individuals with better size-comparison span and memory updating performance had better recall of the cue words. Better reading span performance but smaller size-comparison span performance were furthermore associated with larger peak pupil dilation during listening, as shown using a linear regression model. This may suggest that better ability to suppress irrelevant information tapped by the size-comparison span test can reduce cognitive load during a combined listening and memory task, while larger working memory capacity allows better recall but with greater associated cognitive load.

## ACKNOWLEDGMENTS

This work is supported by grants from The Swedish Research Council. We thank Hans van Beek for his technical support in the data collection.
